# Medical History from the Earliest Times

**Published:** 1892-12-17

**Authors:** E. T. Withington


### Medical History from the Earliest Times.

By E. T. Withington, M.A., M.B. Oxon.

### XXXVI.—THE SCHOOL OF SALERNO.

Salerno, or Salernum, a town beautifully situated on the Italian coast, about thirty miles south-east of Naples, was, even in the time of Horace, a favourite health resort of the Romans, its air being considered more bracing than that of Baiæ. In Christian days it became yet more attractive to the sick, for its cathedral contained what were believed to be the wonder-working relics of St. Matthew; and if three other shrines claimed the head, and seven the body of the Evangelist, the bones of the holy virgins, Theckla, Susannah, and Archelais, were unique and hardly less effective.

Where patients congregated doctors would naturally follow. In the ninth century we already hear of distinguished Salernitan physicians; in the tenth we find one of them at the French court, while bishops and nobles travel from great distances to Salerno for medical aid; and during the next two centuries the fame of "the Hippocratic city" spread throughout Europe. But its decline was equally rapid, and when, in 1811, an edict of Napoleon put an end to the most ancient of universities, even professed historians knew little more than that it had been a great medical school founded by Charlemagne, or the Saracens, or Constantine the African, or, at any rate, by the Benedictine monks. The accidental discovery at Breslau, in 1837, of thirty-five manuscripts by distinguished Salernitan masters, encouraged and assisted further investigation, and the following is a brief outline of our present knowledge.

Only the last of the above theories as to the origin of the school deserves consideration, and among many arguments which conclusively prove that it was not a monastic but a secular institution, we need only mention that Jews and women were connected with it from early times, and that many of its professors were married, and some rich, and could therefore be bound neither by vows of chastity nor poverty. Law and philosophy were soon added to medicine, but theology, if ever taught at all, was the last of the faculties to complete the "Studium generale," or University of Salerno. The ancient chronicles of the city declare that the school was founded by four masters, Abdallah (Adala) the Arab, Eli (Elinus) the Jew, Pontus the Greek, and Salernus the Latin, who lectured to their pupils each in his own tongue. It would be pleasant to believe that representatives of the four divisions of civilised man, then persecuting and warring upon each other in the name of religion, had met together in this beautiful spot for the peaceful study of our sacred art. But the story is clearly mythical. Abdallah had but lately issued from his deserts when the school originated, and he would have found Salerno an unpleasant abode at any time, for it was first an outpost of Christianity against the Saracens, and afterwards a favourite halting place for crusaders. The others, however, may be accepted, at least allegorically. The earliest known Salernitan doctors bear the Jewish names Joseph and Joshua: numerous Greeks, as we have already seen, came to Salerno during the iconoclast persecution, and the formation of the school may not unreasonably be attributed to the combination of these elements with the remains of an ancient Latin "collegium medicorum."

The golden age of Salerno lasted from A.D. 1000 to 1200, and these two centuries form two epochs roughly divided by the interesting episode of Constantine the African. The first period is distinguished by the names of Gariopontus, the elder and younger Platearius, and the elder and younger Copho. Gariopontus, who seems to have been a Greek, wrote the "Passionarius," a medical compendium in which the doctrines of the Methodists are combined with prescriptions taken from Galen, Alexander, Paulus, and the Empiric writers already mentioned. The younger Platearius was the author of the "Practica," a collection of notes on treatment in which he often quotes his father's opinions and practice, while to one of the Cophos is due the first modern anatomical treatise, the "De Anatome Porci," a work describing the shape and position of the viscera of the pig, which animal, says the author, resembles man internally just as the monkey resembles him externally. At the close of the century appeared the most famous product of the school, the "Regimen Sanitatis," "Schola Salernitana," or "Flos Medicinæ," as it is variously called, a medical poem which went through more than 240 editions, and was translated into nearly every civilised language. It consisted originally of about 360 irregularly rhymed hexameters, or "Leonine" verses, and was dedicated to Robert of Normandy, son of the Conqueror, who stayed at Salerno to be healed of a wound in his arm. In it we find the entire popular and proverbial medicine of the age, some of which still survives. Here, for instance, is the politer original of Longfellow's "Joy, temperance, and repose, slam the door on the doctor's nose."

> Si tibi deficiant medici, medici tibi fiant,
> Haec tria, mens hilaris, requies, moderata diæta.

The virtue of cleanliness is highly valued: "Si fore vis sanus, ablue sæpe manus," and nothing could be better than the advice, "Non bibe ni sitias, et non comedas saturatus."

Constantine the African, "Orientis et Occidentis Magister, novusque effulgens Hippocrates," as his admiring biographer calls him, came to Salerno about 1075, bringing with him all the learning of the East, but he soon retired to the monastery of Monte Cassino, where he wrote medical works, which still further increased his renown till they were found to be for the most part translations from Isaac Judeus and Haly Abbas, those parts only being omitted which might betray their Arabic origin. It is, however, not impossible that Constantine acted under pressure from his abbot, Desiderius, afterwards Pope Victor III., who may have preferred a slight sacrifice of truth to the scandal of permitting avowed translations from paynim writers to issue from his monastery.

Constantine's influence upon the school does not appear to have been great, for the Salernitans prided themselves on being the direct inheritors of the classical physicians, and refused to accept the Arabic doctrines even in a concealed form. But they adopted many of the new Eastern drugs, and the chief effect of Constantine's translations is seen in the great attention paid to materia medica during the twelfth century. The works of Nicholas Praepositus (1100) and Matthew Platearius (1140) formed the pharmacopœias of the middle ages, and in the "Circa instans" of the latter we find the first Latin descriptions of two interesting, though very different drugs, mercury and mumia. "Mercury (he declares) is hot and moist in the fourth degree, but some say it is cold in the fourth degree. It is used in ointments for scabies, pediculi capitis, and eruptions on the face; its fumes are highly injurious, and may cause paralysis and even death." "Mumia, according to Constantine, is hot and dry in the fourth degree. It is found in tombs, for the ancients preserved their dead with balsam and myrrh, as is still done by the heathens of Babylon. Mumia acts as an astringent in haemorrhage and dysentery, and assists the healing of ulcers."

Commentaries on the Salernitan pharmacopœias were written by two French members of the school, Bernard of Provence, and Gilles (Aegidius) of Corbeil, afterwards physician to Philip Augustus, who both studied there towards the close of the twelfth century. The former gives us many curious glimpses of the beliefs and practices of the age. "Antimony with raw egg is good for cough. The Salernitan ladies fumigate themselves with antimony." "Coral.—If anyone has palpitation at night, which often occurs after hot food, let him put a coral in his mouth, and he will be at once cured. This is why the Hospitallers and Templars carry a piece of coral in their belts." "Cubebs cure drunkenness, and prevent the breath smelling the next morning; never take more than five berries at once." "Cyclamen.—I, Magister Bernardus, of Provence, gave the juice of cyclamen to a friend with a quartan ague, and by God's grace he was cured." "Warmth is the principle of life. My master, Salernus, cured his squire (armigerum), who was nearly killed by a fall, by burying him in a dung-heap up to his neck. He would also have recovered had he been put in the belly of a recently-killed animal, such as an ox or horse." "If a man is bitten by a mad dog, immediately put some of its hair on the bite, or make the dog, or some part of him, into a plaster."

